# Effects of Oxygen Transference on Protease Production by *Rhodotorula mucilaginosa* CBMAI 1528 in a Stirred Tank Bioreactor

**DOI:** 10.3390/bioengineering9110694

**Published:** 2022-11-15

**Authors:** Suellen Machado, Valker Feitosa, Omar Pillaca-Pullo, Luciana Lario, Lara Sette, Adalberto Pessoa, Harley Alves

**Affiliations:** 1 Departamento de Farmácia, Programa de Pós-Graduação em Ciências Farmacêuticas, Universidade Estadual da Paraíba (UEPB), Campina Grande 58429-500, Brazil; 2 Departamento de Tecnologia Bioquímico-Farmacêutica, Universidade de São Paulo (USP), São Paulo 05508-000, Brazil; 3 Faculdade de Biomedicina, Instituto de Ciências Biológicas, Universidade Federal do Pará (UFPA), Belém 66077-830, Brazil; 4 Departamento de Medicina e Enfermagem, Universidade Federal de Viçosa (UFV), Viçosa 36570-900, Brazil; 5 Centro de Investigación en Biodiversidad para la Salud, Universidad Privada Norbert Wiener, Lima 15046, Peru; 6 Instituto de Ingeniería Ambiental, Química y Biotecnología Aplicada (INGEBIO), Facultad de Química e Ingeniería del Rosario, Pontificia Universidad Católica Argentina (UCA), Rosario 2000, Argentina; 7 Departamento de Biologia Geral e Aplicada, Universidade Estadual Paulista Júlio de Mesquita Filho (UNESP), Rio Claro 13506-900, Brazil

**Keywords:** extremophilic, psychrophilic, proteinase, proteolytic, bioreactor, oxygen transference

## Abstract

Microbial proteases, especially aspartic proteases, are an essential group of enzymes produced from different microorganisms. Microbial proteases have several applications, mainly in the food, beverage, cosmetic, and pharmaceutical industries, due to their efficiency in the processing and in the manufacturing stages. The yeast *Rhodotorula mucilaginosa* CBMAI 1528 was isolated from the Antarctic environment and was previously reported to have higher extracellular aspartic protease production. In addition, advances in the operational conditions of bioreactors for enzyme production are important to reduce the gap associated with scaling−up processes. This is the first study that evaluates the influence of oxygen transference (*k_L_a*) on the protease production of *R. mucilaginosa* yeast. To that end, batch cultures were created in a stirred tank bioreactor using Sabouraud dextrose broth at 25 °C for 72 h under *k_L_a* values from 18 to 135 h^−1^. The results show that *k_L_a* (121 h^−1^) obtained at 500 rpm and 1.5 vvm plays an important role in protease production (124.9 U/mL) and productivity (6.784 U/L.h) as well as biomass (10.4 g/L), *μ_max_* (0.14 h^−1^) and *Y_x/s_* (0.484 g/g). In conclusion, *R. mucilaginosa* showed high yield production in aerobic culture with the efficiency of protease expression and secretion influenced by *k_L_a*. In this sense, our results could be used for further industrial investment.

## 1. Introduction

Proteases, proteinase, or proteolytic enzymes (E.C. 3.4) are widely used for the catalytic hydrolysis of peptide bonds and for breaking down proteins whose specificity is related to the amino acid sequence and position of the peptide bond. Despite being present in plants, animals, and humans, native proteases are predominantly sourced from microbial species [1]. Proteases have numerous biotechnological applications, including in detergent and cleaning products, in leather and textile, in biofuel, in bioremediation, in pulp and paper, in food and beverages, and in forage and animal feed, as well as in chemical, cosmetic, and pharmaceutical fields [2,3]. The global market of enzymes was valued at USD 11.47 billion in 2021 and is projected to expand at a compound annual growth rate of 6.5% until 2030. This market is represented by carbohydrate, protease, lipase, polymerase, and nuclease. The demand for protease is growing significantly across the North American region (37.5% of the global revenue in 2021), mainly due to the growth in pharmaceutical and food and beverage industries [4].

Microbial proteases represent two-thirds of commercial proteases worldwide [5]. Microorganisms are preferred over the others for the large-scale production of many enzymes for the following reasons: they are present everywhere in nature; they can be produced in relatively higher quantities than in plants and in animals; they grow quickly and simply; they have unique physiological and biochemical properties, as well as simple culture conditions; they are produced by cells of easy manipulation [6,7]; and they are secreted from microorganisms, facilitating the down streaming stages. Moreover, the proteolytic enzymes found in microbes are small, dense, and structurally spherical and have many applications in various industrial sectors [5].

Several microorganisms have been investigated in the search for new isolates that are good producers of proteases [6]. Among the extremophilic microbial community, cold-adapted microorganisms (i.e., psychrophilic and psychrotolerant) comprise the most explored groups for biotechnological and industrial applications. Diverse cold-active proteases have been isolated from bacteria [8], but, to date, only a few fungal proteases have been reported, such as *Vanrija humicola* [9] and *Glaciozyma antarcticum* [10].

Unique environments, such as Antarctica, are ecological niches for taxonomically, physiologically, and phylogenetically uncommon microorganisms. Thus, microorganisms that inhabit cold environments must adapt to harsh conditions (e.g., low temperatures, osmotic stress, and high UV radiation). They have been used in the bioprospecting of enzymes, such as proteases. Considering their unique properties, cold-active enzymes could be applied to industrial and environmental processes carried out at low or mild temperatures (i.e., 10−70 °C) [11], thus reducing energy consumption and the wear and tear of bioprocesses compared to mesophilic and thermophilic enzymes [8,12].

Hence, our group isolated *Rhodotorula mucilaginosa* CBMAI 1528 (= L7 and CRM 669) from the Antarctic continent, which was shown to be a great producer of extracellular protease [13]. Chaud et al. evaluated the effect of nutrient medium components (e.g., peptone and rice bran extract), pH, and temperature on the extracellular proteolytic activity of *R. mucilaginosa* cultured in a rotary shaker [14]. The protease was purified using liquid–liquid extraction [15] and CM-Sepharose cation exchange chromatography, and then characterized as a monomeric 34.5-kDa protein with optimal catalytic activity at pH 5.0 and 50 °C stable with NaCl [16]. In addition, amino acid sequencing by mass spectrometry revealed that this enzyme is an aspartic protease belonging to the pepsin family and peptidase A1 subfamily, once proteolytic activity decreases 95 % by pepstatin A, a specific inhibitor of aspartic acid proteases [16].

Aspartic proteases (EC 3.4.23), aspartyl proteinases, or acidic proteases are endopeptidases with two aspartic acid residues within their active site that are vital for their catalytic activity. Hence, enzymes from microbial sources are categorized, such as pepsin-like enzymes and rennin-like enzymes [17]. Aspartic proteases present well-established applications in the development of traditional and novel food products. They are also extensively used in cheese manufacturing (e.g., milk-clotting agents), beverage processing (e.g., clarification and preservation) [18], and in the pharmaceutical industry (e.g., in digestive aids and the treatment of certain lytic enzyme deficiency syndromes) [19].

The genus *Rhodotorula* includes species belonging to the Basidiomycota division, in particular, the pink-pigmented species found and isolated from different sources. Extensive literature can be found on their natural ability to produce enzymes in the rotatory shaker; nevertheless, there are few reports on enzyme production by *R. mucilaginosa* strains in bioreactorsusing different processes such as aeration, agitation, oxygen transfer, carbon and nitrogen sources, temperature, and pH. Furthermore, the production of lipids [20,21,22] and carotenoids [23] in bioreactors has been reported. 

As a matter of fact, the species *R. mucilaginosa* has received increasing attention due to its ability to grow in extreme ecosystems and its natural capability to produce valuable compounds of industrial interest such as lipids [20,21,22], carotenoids [23], acetylxylan esterase [24], epoxide hydrolase [25], serine protease [26], neutral and acid proteases [27], esterase [28], lipases [29,30,31], phenylalanine ammonia-lyase [32], pectinases [33,34], cutinase [35], phytase [36], glycosidase [37], and aldolase [38]. Optimal bioprocess conditions are crucial for the production of each enzyme in commercial practice. In this sense, aerobic bioprocess development using *R. mucilaginosa* should be carried out to avoiding oxygen limitation. The evaluation of constant volumetric mass transfer coefficient (*k_L_a*) is an important step in order to supply adequate oxygen transfer, mixing/sparging operation, and scaling-up criterion [39,40] due to effects in physical and biological characteristics related with metabolic pathways for growing and product formation [41,42]. Thus, the aim of this work was to investigate the effect of the volumetric oxygen mass transfer coefficient (*k_L_a*) on aspartic protease production during the cultivation of marine Antarctic *R. mucilaginosa* CBMAI 1528 in a stirred tank bioreactor.

## 2. Materials and Methods

### 2.1. Microorganism and Growth Conditions

*R. mucilaginosa* (original code L7) was isolated from a marine alga collected in the Antarctic continent and identified through the similarity of the partial 26S rDNA gene [13]. The strain was deposited in the Brazilian Collection of Environmental and Industrial Microorganisms (CBMAI) under the acronym CBMAI 1528 and in the UNESP Microbial Resources Center (CRM-UNESP) under the acronym CRM 669. The strain was grown on Sabouraud dextrose broth (40 g/L dextrose, 10 g/L peptone, pH 5.6) (BD Biosciences, San Jose, CA, USA) at 25 °C, under orbital shaking (150 rpm) for 24 h. The yeast strain was stored in Sabouraud dextrose broth with glycerol (20% wt) at –70 °C [16].

### 2.2. Inoculum and Culture Conditions

The inoculum was obtained by transferring 1 mL of stock culture to 250 mL Erlenmeyer flasks containing 50 mL of Sabouraud dextrose broth followed by incubation at 25 °C, 180 rpm, for 18 h under orbital shaking [43]. Batch cultures were created in 3.0 L bench-top stirred tank bioreactors (BioFlo 110 and 115, New Brunswick, Edison, NJ, USA) with a 2.0 L working volume. Thus, 200 mL of the inoculum was added to 1.8 L Sabouraud dextrose broth (previously autoclaved at 121 °C for 20 min) with 0.002% Y-30 antifoam emulsion (Sigma-Aldrich, Saint Louis, MO, USA). The bioreactor was equipped with a thermometer, pH sensor, dissolved oxygen sensor, tachometer, air-flow meter, internal pressure sensor, and foam-sensing probe.

During the experiments, the temperature was maintained constant at 25 °C by a heating system in the bottom and cooling water. The dissolved oxygen concentration and pH were measured by electronic probes (Mettler Toledo, Greifensee, Switzerland), and filtered air was continuously bubbled into the medium through a multipoint sparger. The yeast was cultivated for 72 h without pH control. The cultures were carried out in different agitation (rpm) and aeration (vvm), and six *k_L_a* values were obtained according to Table 1.

### 2.3. Quantification of Biomass, Glucose, Total Protein, and Proteolytic Activity

The samples of 5 mL were collected from each culture for quantification. The biomass concentration was gravimetrically determined, the cell was removed by centrifugation at 4000× *g* for 10 min, and the pellet was dried at 60 °C in an oven until it reached a constant weight. The biomass concentration was expressed in grams of dry cells per liter of cultivation medium (g/L).

The glucose concentration was determined by spectrophotometry by measuring the absorbance at 500 nm according to the glucose oxidase method (Laborclin, Pinhais, PR, Brazil). 

The total protein content was measured by the bicinchoninic acid (BCA) (Sigma-Aldrich, Saint Louis, MO, USA). The samples were collected, centrifuged, and diluted in phosphate-buffered saline (PBS) at 1:20 (sample:buffer). The diluted samples were incubated with BCA solution in a 96-well microplate, with 25 µL of sample to 200 µL of the reagent. The plate was incubated at 37 °C for 30 min. The UV/Vis measurements were performed in a microplate spectrophotometer at 562 nm.

The proteolytic activity was determined by the digestion of azocasein (Sigma-Aldrich, Saint Louis, MO, USA). Culture supernatant (150 µL) was incubated with 150 mL of 0.5% azocasein (Sigma-Aldrich, Saint Louis, MO, USA) in 50 mM of sodium acetate buffer (pH 5.0) for 40 min at 37 °C. The reaction was stopped by adding 150 µL of 10% (*w*/*v*) trichloroacetic acid. After centrifugation of the reaction mixture, 100 μL of the supernatant was mixed with 100 μL of 0.5 M KOH, and the absorbance at 430 nm was measured. The samples were assayed in three independent measurements, and the activity was expressed as units of enzyme activity (U). One U was defined as the amount of enzyme leading to a 0.001 increase in the absorbance under the assayed conditions. 

### 2.4. Determination of Volumetric Oxygen Transfer Coefficient (k_L_a)

The values of the initial volumetric oxygen transfer coefficients (*k_L_a*) were determined in distilled water at 25 °C, using the static gassing-out method described by Pirt [44]. This method estimates *k_L_a* values based on the oxygen dissolution rate as a function of agitation and aeration conditions. Nitrogen gas was injected through the air sparger until it reached a deoxygenated state. Then, the air supply began to replace the nitrogen, and the increase in the rate of dissolved oxygen concentration in the water was measured [45]. The mass balance for the dissolved oxygen in the well-mixed liquid phase can be described through the conventional Pirt’s mathematical model as:

(1) $$\frac{dC_{}}{dt}=k_{L}a\left(Cs-C\right)$$

where *dC/dt* is the rate of O_2_ accumulation in the liquid phase, *k_L_a* is the volumetric mass transfer coefficient (h^−1^), (*Cs − C*) is the driving force causing the mass transfer, and *C_S_* and *C* refer to the liquid-phase oxygen concentration at saturation at any time, respectively.

### 2.5. Kinetic Parameters Calculation

The maximum specific growth rate (*µ_max_*) (2) and the substrate-to-cell conversion factor (*Y_X/S_*) (3) were calculated according to equations reported by Pillaca-Pullo et al. [46]:

(2) $$\mu_{max}=\frac{1}{\left(t_{f}-t_{0}\right)}ln\frac{X_{f}}{X_{0}}$$

(3) $$Y_{\frac{X}{S}}=\frac{\left(X_{f}-X_{0}\right)}{\left(S_{f}-S_{0}\right)}$$

where *X_f_* is the cell concentration during the exponential phase, *X*_0_ is the initial cell concentration, and *t_f_* and *t*_0_ are the final and initial time, respectively. *X_max_* is the maximum cell concentration, *X*_0_ is the initial cell concentration, and *S*_0_ and *S_f_* are the initial and final glucose concentration, respectively.

The enzyme productivity (*PrP*) (4) was calculated according to the following equation:

(4) $$P_{r}P=\frac{\left(P_{f}-P_{0}\right)}{\left(t_{FP}-t_{0}\right)}$$

where *P_f_* and *P*_0_ are proteolytic final and initial activities, respectively, *t_FP_* is the time corresponding to the cultivation at *P_f_*, and *t*_0_ is the initial time (zero).

## 3. Results

Bioprocesses for protease production were carried out in stirred tank bioreactors under agitation ranging from 100 to 500 rpm and aeration ranging from 1.0 to 2.5 vvm. To better evaluate the influence of the oxygen supply, the *k_L_a* was determined, as shown in Table 1, ranging from 18 to 135 h^−1^.

**Figure 1:**
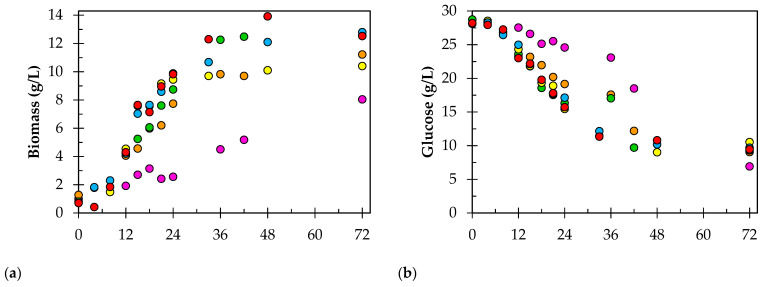

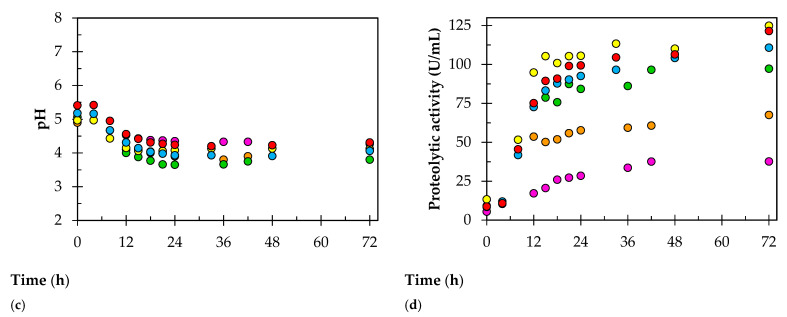
Representation of biomass concentration (**a**), glucose consumption (**b**), pH (**c**), and proteolytic activity (**d**) curves of *R. mucilaginosa* CBMAI 1528 in bioreactor under different *k_L_a* conditions: 18 h^−1^ (purple, run 1), 49 h^−1^ (orange run 2), 99 h^−1^ (green, run 3), 121 h^−1^ (yellow, run 4), 135 h^−1^ (blue, run 5), and 102 h^−1^ (red, run 6).

Figure 1 and Table 2 summarize the final values of the cell growth (biomass), substrate (glucose and total proteins), pH, and proteolytic activity of all bioprocesses. Experimental runs 1, 2, and 3 were conducted using aeration of 1.0 vvm, and agitation varied at 100, 300, and 500 rpm. Proteolytic activity increased (37.7, 67.6, and 97.2 U/mL, respectively), while agitation also increased. For the subsequent experiments, the agitation was set at 500 rpm, and the aeration was varied. Experimental runs 4, 5, and 6 were performed with 1.5, 2.0, and 2.5 vvm, respectively. However, the proteolytic activity found for run 4 (*k_L_a* 121 h^−1^) seems to be on the same level as the values for runs 5 and 6 (124.9, 110.8, and 121.4 U/mL, respectively). This fact suggests that the supply of oxygen, which results from agitation and aeration, becomes indifferent to the value of the proteolytic activity, i.e., the medium appears to be saturated with oxygen. As greater amounts of air volume are added, there is little difference in the values for activity.

*R. mucilaginosa* is an aerobic microorganism, therefore it requires the provision of oxygen [47]. Aeration and agitation of the growth medium are essential for successful fermentation and could be beneficial to the growth and performance of microbial cells by improving mass transfer characteristics concerning substrate, product, and oxygen. The yeast produces carotenoids located in the cell wall [48], hence the pink color that characterizes the yeast, which becomes a visual indicator of cell growth (data not shown). The results showed that the maximum biomass accumulation (12.8 g.L^−1^) was observed at run 5 (*k_L_a* 135 h^−1^); however, there was not much difference in the growth at different aeration rates in experimental run 3 (*k_L_a* 99 h^−1^) and run 6 (*k_L_a* 102 h^−1^).

The data obtained from the calculation of the productivity related to protease production with culture time are presented in Table 3. Experimental run 4 achieved higher proteolytic activity (124.9 U.mL^−1^) at 72 h, but the highest productivity occurred at 12 h, when the activity reached 94.8 U.mL^−1^ (data not shown). From the industrial viewpoint, in order to reach a shorter time for production, it becomes more advantageous to conduct six sequential or simultaneous processes over 12 h, instead of a single batch for 72 h, considering that the proteolytic activity will be ~4.5-fold higher in the first scenario. Therefore, it is crucial to find an experimental condition that promotes increased production and is also industrially advantageous.

For conversion yields (*Y*), the variables must be in the same unit of measurement (in this case, g.g^−1^). The factor *Yx/s* is the substrate conversion cells. The *μ_max_* values (i.e., the maximum specific growth rate) and *Yx/s* for each experiment are reported in Table 4.

The cell growth rate becomes a determining factor for the efficiency of the process when there are problems with oxygen transfer to the culture medium. Ideally, the growth rate reaches desirable levels to obtain high cell concentrations and increase the amount of product formed. The highest value for *μ_máx_* was found in run 4 (*k_L_a* 121 h^−1^), with 0.14 h^−1^, between 4 and 15 h of cultivation. The lowest value was found in run 1, with 0.06 h^−1^. In practical terms, this means that experimental runs 3, 5, and 6 have the same biomass after 72 h of cultivation (12.56, 12.83, and 12.52 g.L^−1^, respectively). A higher *Yx/s* 0.63 g.g^−1^ was found for run 6 (*k_L_a* 102 h^−1^), which means that 63% of glucose was used to form the biomass. The lowest value was obtained for run 1 (*k_L_a* 18 h^−1^), in which only 29% of glucose was converted to the cells, showing that *k_L_a* influenced *Yx/s* positively.

## 4. Discussion

Bioprocesses can be carried out in three scales (bench, pilot, and manufacturing). In the case of enzyme production, large-scale production is preferred using bioreactors. However, this production is first established at the laboratory level to reach a larger scale under equal or improved yield [46]. The stage for establishing conditions can be carried out in shake flasks or bioreactors, despite the fact that the physical and biological factors would be different in these systems. The selection of design conditions and operational procedures is very important to expand the process and to ensure that the effect of the variables on the process are the same [40]. The scaling up of the fermentation processes from the laboratory-scale to commercial units is challenging due to the difficulty in assessing the factors of influence during cultivation [49]. It is well known that microorganisms are more susceptible to large-scale environmental variables. According to Mussagy et al. [50], several factors such as medium composition, pH, temperature, aeration, and agitation influence microbial metabolite production and cell growth. The genus *Rhodotorula* includes species belonging to the Basidiomycota division, in particular the pink-pigmented species found and isolated from different sources. Extensive literature can be found on their natural ability to produce enzymes in the rotatory shaker, as shown in Table 5. Nevertheless, there are few reports on the enzyme production by *R. mucilaginosa* strains in bioreactors, including phenylalanine ammonia-lyase [32], lipase [30], and esterase [28], carried out under parameters such as aeration, agitation, *k_L_a*, carbon and nitrogen sources, temperature, and pH, as summarized in Table 6. Furthermore, the production of carotenoids [23] and lipids [20,21,22] in bioreactors has been reported. Notwithstanding, the scarcity of specific literature relating to the influence of *k_L_a* on growth and protease production by *R. mucilaginosa* using bioreactors limits the direct comparison of the present results.

Assessing the 11.25-fold increase in proteolytic activity, from 11.1 U.mL^−1^ in the rotary shaker [13] to 124.9 U.mL^−1^ on the bioreactor scale at *k_L_a* 121 h^−1^, the supply of oxygen to the production was essential to increase the extracellular protease production of the yeast *R. mucilaginosa* CBMAI 1528, since the protease production increased considerably in the bioreactor. According to Fenice et al. [51], considering a single parameter, agitations was more effective than aeration for enzyme production because the relative growth curve under agitation was steeper. In fact, high agitation generates bubbles that increase the gas–liquid interface area and the residence time in the medium culture, which causes a higher rate of dissolved oxygen [52]. The *k_L_a* measures the oxygen transfer performance from the gaseous to the liquid phase. Thus, oxygen is essential for synthesizing biomass and enzymes, since it is involved in the metabolism of the microorganism [53]. Aeration supplies the necessary oxygen for cell growth and eliminates the exhausted gas generated during the bioprocess [54]. In the present study, the biomass values increased when aeration was fixed (1.0 vvm), whereas cell growth decreased (run 4, *k_L_a* 121 h^−1^) or remained constant (runs 5 and 6, *k_L_a* 135 and 102 h^−1^, respectively) when the aeration values increased. In fact, shear stress, as oxygen supply, can be very harmful and plays an important role in the organism’s morphology/physiology and, consequently, in biomass formation and enzyme production [55,56]. 

The highest value of protease production at *k_L_a* 121 h^−1^ can be attributed to the increased oxygen availability in the culture medium. However, higher aeration rates can increase the oxygen system pressure without increasing production. Several studies show that aeration and agitation affect the dissolved oxygen concentration in the culture medium, thus enhancing the biological and physical characteristics associated with both growth and enzyme production [57,58]. For aerobic processes, oxygen transfer is a key variable and is a function of aeration and agitation. These parameters not only affect the productivity of the microbial process, but also the overall energy required by the production process [29], since the metabolic fluxes correlated to product formation can be influenced by the oxygen level [41,42]. For example, oxygen transfer was a critical parameter for maximum lipase production by *R. mucilaginosa* MTCC 8737, which can be achieved by combining aeration and agitation in a bioreactor [30]. 

Proteolytic activity increases with agitation, but is indifferent to higher aeration. This result was expected, because stirred tank bioreactors provide the efficient mixing associated with a high transference of heat, substrates, and oxygen [42]. To describe the oxygen supply condition of a fermentation system, *k_L_a* is commonly used as a parameter implied in the bioreactors’ mixing–sparging equipment [39]. The *k_L_a* is the most significant parameter to measure transfer phenomena, including oxygen transfer, inside a bioreactor [53]. Its values are affected by many factors, such as the bioreactor design, medium formulation, medium strength, aeration with sparger, and agitation [59].

Abdella et al. [60] reported that a high *k_L_a* was preferred in the xylanase production by a recombinant *Aspergillus nidulans* strain. Notwithstanding, a high agitation rate harmed enzyme production due to high shear stress on the production organism. Fenice et al. [51] also correlated the reduction of chitinolytic activity to the increase of aeration: the lowest activity (92 U.L^−1^) was obtained at 0.5 vvm both at 200 and 500 rpm (*k_L_a* 18 and 51 h^−1^, respectively), whereas the highest enzyme activity (383.7 U.L^−1^) was achieved at 1.0 vvm and 300 rpm (*k_L_a* 151 h^−1^).

## 5. Conclusions

Reports on aspartic proteases from cold-adapted yeasts are scarce. Based on our results, the production of an aspartic protease by *R. mucilaginosa* CBMAI 1528 depends on oxygen transference in stirred tank bioreactor. Higher proteolytic activity was found at a *k_L_a* 121 h^−1^. Hence, this yeast followed an aerobic culture, which makes parameters such as aeration and agitation essential for growth and protease expression and secretion. Further assays should focus on the scaling-up process using the *k_L_a* criterion.

## Figures and Tables

**Table 1:** Agitation (rpm), aeration (vvm) conditions, and experimental results of the volumetric oxygen transfer coefficient (*k_L_a*) for *R. mucilaginosa* CBMAI 1528 cultivation in bioreactor.

Experiment	Agitation (rpm)	Aeration (vvm)	*k_L_a* (h^−1^)
1	100	1.0	18
2	300	1.0	49
3	500	1.0	99
4	500	1.5	121
5	500	2.0	135
6	500	2.5	102

**Table 2:** Experimental values of biomass concentration, glucose, total proteins, pH, and proteolytic activity of *R. mucilaginosa* CBMAI 1528 cultivation in bioreactor for 72 h.

Parameter	Experiment (*k_L_a*)					
	1 (18 h^−1^)	2 (49 h^−1^)	3 (99 h^−1^)	4 (121 h^−1^)	5 (135 h^−1^)	6 (102 h^−1^)
Agitation (rpm)	100	300	500	500	500	500
Aeration (vvm)	1.0	1.0	1.0	1.5	2.0	2.5
Biomass (g.L^−1^) ± sd	8.0 ± 0.3	11.2 ± 0.5	12.6 ± 0.2	10.4 ± 0.6	12.8 ± 0.4	12.5 ± 0.8
pH	4.3	3.8	3.8	4.1	4.0	4.3
Proteolytic activity (U.mL^−1^) ± sd	37.7 ± 7.1	67.6 ± 5.3	97.2 ± 1.3	124.9 ± 5.1	110.8 ± 3.4	121.5 ± 2.5
Glucose (g.L^−1^) ± sd	6.9 ± 0.3	9.0 ± 0.8	9.3 ± 0.5	10.5 ± 1.2	9.7 ± 0.7	9.5 ± 1.1
Total protein (g.L^−1^) ± sd	3.8 ± 0.1	3.6 ± 0.3	3.4 ± 0.1	4.0 ± 0.2	4.6 ± 0.4	4.2 ± 0.2

**Table 3:** Productivity in protease activity ± SD during *R. mucilaginosa* CBMAI 1528 cultivation in bioreactor up to 72 h.

Time (h)	Experiment (*k_L_a*) / Productivity (U/L.h^−1^)					
	1 (18 h^−1^)	2 (49 h^−1^)	3 (99 h^−1^)	4 (121 h^−1^)	5 (135 h^−1^)	6 (102 h^−1^)
4	nc	nc	nc	nc	0.813 ± 0.033	0.519 ± 0.031
8	nc	nc	nc	4.784 ± 0.012	4.144 ± 0.010	4.598 ± 0.014
12	0.977 ± 0.023	3.764 ± 0.031	5.488 ± 0.012	6.784 ± 0.023	5.327 ± 0.010	5.535 ± 0.019
15	1.009 ± 0.020	2.781 ± 0.019	4.639 ± 0.014	6.132 ± 0.019	4.967 ± 0.021	5.378 ± 0.010
18	1.139 ± 0.011	2.411 ± 0.020	3.699 ± 0.023	4.861 ± 0.010	4.394 ± 0.024	4.563 ± 0.022
21	1.039 ± 0.019	2.255 ± 0.022	3.728 ± 0.021	4.377 ± 0.010	3.884 ± 0.011	4.299 ± 0.020
24	0.960 ± 0.038	2.049 ± 0.012	3.131 ± 0.011	3.841 ± 0.034	3.494 ± 0.023	3.776 ± 0.011
33	nc	nc	nc	3.030 ± 0.024	2.663 ± 0.012	2.902 ± 0.010
36	0.782 ± 0.021	1.414 ± 0.011	2.138 ± 0.028	nc	nc	nc
42	0.765 ± 0.014	1.244 ± 0.028	2.081 ± 0.024	nc	nc	nc
48	nc	nc	nc	2.016 ± 0.011	1.989 ± 0.026	2.035 ± 0.012
72	0.447 ± 0.030	0.820 ± 0.019	1.223 ± 0.014	1.549 ± 0.010	1.417 ± 0.020	1.565 ± 0.023

nc = no calculated.

**Table 4:** Parameter values of *R. mucilaginosa* CBMAI 1528 cultivation carried out in bioreactor under different *k_L_a* values.

Parameter	Experiment (*k_L_a*)					
	1 (18 h^−1^)	2 (49 h^−1^)	3 (99 h^−1^)	4 (121 h^−1^)	5 (135 h^−1^)	6 (102 h^−1^)
*µ_máx_* (h^−1^)	0.06	0.08	0.12	0.14	0.12	0.13
*Y_x/s_* (g.g^−1^)	0.29	0.52	0.61	0.48	0.54	0.63

**Table 5:** Enzyme production by *Rhodotorula mucilaginosa* strains in agar plate or rotatory shaker.

Enzyme	Strain	Maximum Activity	Time (h)	rpm	T (°C)	Main Nutrients	X (g.L^−1^)	Initial pH	Final pH	Ref.
Acetylxylan esterase	NRC 211003	2.1 µmol/mL.h	120	200	30	(NH_4_)_2_SO_4_, glycerol	nr	5.5	nr	[24]
Epoxide hydrolase	M002	nr	nr	nr	nr	nr	nr	nr	nr	[25]
Serine protease	nr	nr	24	nr	28	Dextrose, peptone, (NH_4_)_2_SO_4_,	nr	5.5	nr	[26]
Aspartic protease	CBMAI 1528	11.1 U/mL	120	150	25	Dextrose, animal peptone, casein peptone	nr	5.5	nr	[13]
Aspartic protease	CBMAI 1528	~65 U/mL	48	150	25	Dextrose, peptone	~70 Log CFU/mL	5.6	~3.6	[16]
Aspartic protease	CBMAI 1528	33.4 U/mL	120	150	25	Dextrose, peptone	3 × 108 cells/mL	5.5	nr	[14]
Neutral protease	KKU-M12c	140.3 U/mL	48	120	30	Yeast extract, peptone, dextrose, casein	nr	nr	nr	[27]
Acid protease	KKU-M12c	175 U/mL	48	120	30	Yeast Extract, peptone, dextrose, casein	nr	nr	nr	[27]
Lipase	MTCC-8737	29.9 U/L	120	150	28	Dextrose, malt extract, yeast extract, peptone	0.14	nr	nr	[30]
Lipase	P11I89	272.7 U/L	60	200	30	Palm oil, yeast extract, NH_4_NO_3_	11.2	nr	nr	[31]
Pectinase	CRUB138	nr	nr	na	nr	Dextrose, pectin, yeast extract, peptone, agar	nr	7.0	nr	[33]
Pectinase	PT1	400 U/L	nr	150	12	Malt extract, peptone, pectin, K_2_HPO_4_, citrate	nr	5.0	nr	[34]
Cutinase	Pink	9.5 U/mL	96-120	160	30	Lactose, yeast extract,	nr	6.5	nr	[35]
Phytase	JMUY14	205.5 U/mL	168	150	15	Dextrose, peptone, (NH_4_)_2_SO_4_	nr	5.5	nr	[36]
Glycosidase	*nr*	0.42 U/mL	72	nr	nr	nr	nr	nr	nr	[37]

nr = not reported; rpm = agitation; pH = final pH; T = temperature; X = final biomass.

**Table 6:** Biomolecules production by *Rhodotorula mucilaginosa* strains in bioreactor.

Biomolecules	Strain	Maximum Production	Time (h)	Bioreactor	rpm	vvm	*k_L_a* (h^−1^)	T (°C)	Main Nutrients	X (g.L^−1^)	Initial pH	Final pH	Ref.
Aspartic protease	CBMAI 1528	124.9 U/mL	72	STR	500	1.5	135	25	Glucose, animal peptone, casein peptone	10.4	5.5	4.1	This study
Aspartic protease	CBMAI 1528	111.2 U/mL	nr	STR	500	2.0	92	20	Glucose, casein tryptone	6.7	5.6	nr	[43]
Esterase	saar1	19.5 U/mg	20	nr	300	2.5	nr	nr	Dibenzoyl-tartrate, yeast extract, KNO_3_, (NH_4_)_2_SO_4_, NH_4_Cl	nr	7.4	7.7	[28]
Lipase	MTCC 8737	72 U/mL	96	STR	200	2.0	nr	30	Dextrose, malt extract, yeast extract, peptone	6.6	7.0	7.0	[29]
Phenylalanine ammonia-lyase	nr	41 U/g	50	STR	200	1.0	nr	30	Dextrose, peptone, yeast extract, (NH_4_)_2_SO_4_	3.4	6.0–7.0	6.0–7.0	[32]
Lipids	IIPL32	8.6% *w*/*w*	12	Split column airlift	nr	1.5	0.894	32	Sugarcane bagasse	11.6	4.5	4.5	[20]
Lipids	IIPL32	1.83 g/L	nr	STR	180	nr	nr	32	Xylose rich corn cob hydrolysate	nr	5.5	5.5	[21]
Lipids	nr	0.25 g/g	50	STR	300	1.0	nr	28	Glucose, malt extract, peptone	15.0	6.0	6.0	[22]
Carotenoids	MTCC-1403	819.23 µg/g	84	STR	120	1.0	nr	26	Onion peel, mung bean husk	nr	6.2	6.2	[23]

nr = not reported; rpm = agitation; pH = final pH; T = temperature; vvm = aeration; X = final biomass; STR = stirred tank reactor.

## Data Availability

Not applicable.
